# Short-term effectiveness of telehealth interventions for people in maintenance dialysis: differential impacts on clinical outcomes by modality and intervention type - a systematic review and meta-analysis

**DOI:** 10.1186/s12882-026-04833-0

**Published:** 2026-02-20

**Authors:** Xinxia Shao, Lin Qi, Tomoko Kamei

**Affiliations:** 1 Graduate School of Nursing Science Doctoral Course, St. Luke’s International University, 10-1 Akashi-cho, Chuo-ku, Tokyo, 104-0044 Japan; 2 Graduate School of Nursing Science, St. Luke’s International University, Tokyo, Japan

**Keywords:** Telehealth, Maintenance dialysis, Clinical outcomes, Systematic review

## Abstract

**Aim:**

This systematic review and meta-analysis aimed to evaluate the effects of telehealth interventions compared with usual care on clinical indicators during the initial 3 months among adults receiving peritoneal dialysis (PD) or hemodialysis (HD) and to investigate whether different telehealth intervention models produce differentiated outcomes.

**Methods:**

Following PRISMA guidelines, a comprehensive search of eight databases was conducted for randomized controlled trials (RCTs) published until August 2024, involving adult dialysis patients receiving telehealth interventions versus usual care. Outcomes were serum albumin, serum creatinine, hemoglobin, and hematocrit measured at approximately 3 months. Data were analyzed using random-effects models, subgroup analyses, and GRADE for evidence quality assessment.

**Results:**

Five RCTs involving 1455 screened articles were included. Telehealth interventions were associated with changes in serum creatinine in both HD (MD = −80.45; 95% CI [−136.63, −24.27]) and PD patients (MD = −162.67; 95% CI [−193.09, −132.25]). Hemoglobin levels improved notably in PD patients (MD = 8.66; 95% CI [5.89, 11.42]), but not in HD patients. Subgroup analyses showed that education-only interventions increased hemoglobin (MD = 6.68; 95% CI [2.30, 11.05]; I^2^ = 0%; *p* = 0.003). Education-only models indicated short-term improvements in certain laboratory markers; results for education + monitoring were mixed. High heterogeneity (I^2^ ≥ 75%), limited number of studies, small sample sizes, regional restrictions, and low-quality evidence reduced certainty.

**Conclusions:**

Telehealth interventions may provide short-term benefits for specific clinical indicators in maintenance dialysis for PD. Future large-scale, multicenter trials with extended follow-up are needed to clarify optimal telehealth strategies and determine how best to integrate telehealth into routine practice. The study protocol was registered with the International Prospective Register of Systematic Reviews (PROSPERO) at the National Institute for Health Research (CRD: 42024574257).

**Clinical trial number:**

Not applicable.

**Supplementary Information:**

The online version contains supplementary material available at 10.1186/s12882-026-04833-0.

## Introduction

### Clinical impact and implementation challenges of telehealth in maintenance dialysis

Chronic kidney disease (CKD) poses a major global public health challenge [1]. Patients with end-stage kidney disease (ESKD) rely on long-term renal replacement therapies for survival [2]. Maintenance dialysis, including both peritoneal dialysis (PD) and hemodialysis (HD), is a key treatment for patients with ESKD [3, 4].

In recent years, telehealth has emerged as an innovative solution to address the challenges of chronic disease management [5–7]. By using information and communication technologies (ICTs), telehealth enables continuous monitoring, education, and consultation, bringing care into the home and bypassing geographic barriers [8].

Telehealth has demonstrated potential benefits in dialysis, including improved patient satisfaction among PD users [9, 10] and reduced transitions to HD. It has also been associated with fewer hospitalizations and lower hospital admission rates [11–13]. Moreover, telehealth has been shown to reduce the frequency of clinic visits [14], enhance patient satisfaction among HD users [15], and mitigate infection risks [16].

International guidelines designate albumin, creatinine, hemoglobin, and hematocrit as key clinical outcomes that must be routinely monitored in patients receiving maintenance dialysis [17, 18]. These measures offer a sensitive snapshot of nutritional status, muscle mass, anemia control, and dialysis adequacy [4, 17, 18]. Each marker is independently linked to survival. Hypoalbuminemia, and anemia collectively increase mortality risk, while cohort data further show that a declining serum creatinine trajectory predicts poorer outcomes [17, 18].

However, in the dialysis field, existing reviews either focus exclusively on the effects of remote care in PD [11] or explore how telehealth impacts processes and quality of care for patients with end-stage renal disease [19]. There is no comprehensive systematic review dedicated to telehealth in maintenance dialysis populations. Patients starting telehealth face unique challenges that can worsen early instability, such as technological barriers and the difficulties of forming new habits [20, 21], which can each hinder the successful adoption of telehealth interventions. However, limited attention has been given to how these short-term challenges affect key clinical outcomes.

Accordingly, this systematic review and meta-analysis aims to (a) assess the short-term (up to three months) effects of telehealth interventions compared to usual care on albumin, creatinine, hemoglobin, and hematocrit in patients receiving either PD or HD, and (b) explore whether different telehealth intervention models produce varied short-term outcomes within the first 3 months of implementation, thereby guiding tailored strategies for better patient management. This review is intended to provide solid, evidence-based guidance on the best design and implementation of telehealth services for maintenance dialysis populations by addressing those objectives.

## Methods

We conducted this review following the Preferred Reporting Items for Systematic Reviews and Meta-Analyses (PRISMA) statements [22]. The telehealth interventions included methods where healthcare providers, such as nurses and physicians, interacted with patients, provided educational support, or collected and verified information about their physical and mental health using ICTs. The study protocol was registered with the International Prospective Register of Systematic Reviews (PROSPERO) at the National Institute for Health Research (CRD: 42024574257).

### Literature search strategies and sources

Keyword combinations related to telehealth, artificial kidneys, and randomized controlled trials were used (see Appendix S1 in the Supporting Information). Languages were limited to English, Chinese, and Japanese. We conducted a comprehensive literature search of the following databases with no restrictions on publication date: PubMed, CINAHL Ultimate, EMBASE, Cochrane Central Register of Controlled Trials (CENTRAL), Web of Science, Ichushi-web, China National Knowledge Infrastructure (CNKI), and Wan Fang Data. Only peer-reviewed studies were considered. A librarian at our university advised our search strategy. The search covered works published up until August 2024.

### Article selection and parameters

For trials with multiple follow-up assessments, we extracted outcome data at baseline and the assessment closest to 3 months; later follow-up time points were not included in the primary meta-analysis. The inclusion criteria for studies were as follows: (i) Participants—all adult patients (18 years old and above) undergoing maintenance dialysis, including PD or HD; (ii) Intervention—telehealth interventions in which healthcare providers used ICT to interactively communicate with patients, provide educational support, and/or collect and verify patient health information; eligible studies were required to report outcome data at approximately 3 months after intervention initiation (e.g., 10–14 weeks), regardless of the total intervention duration; (iii) Comparator—the control group received only standard regular medical interventions; (iv) Study design—randomized controlled trials (RCTs), including parallel-group RCTs, cluster-RCTs, and crossover trials (considering only data from the first phase before crossover to avoid carryover effects); and (v) Outcomes—included serum albumin, serum creatinine, hemoglobin, and hematocrit at approximately 3 months. The exclusion criteria included those who received inpatient treatment or end-of-life care.

### Selection and data extraction processes

Two researchers (SXX & QL), proficient in Chinese, Japanese, and English, independently conducted data extraction and verification to ensure accuracy and reduce translation bias. We predefined outcomes and extracted outcome data at baseline and the assessment closest to 3 months after intervention initiation.

During the first screening, two researchers (SXX & QL) screened the titles and abstracts of studies to identify studies that met the criteria. During the second screening, independent researchers (SXX & QL) reviewed the full articles and selected those qualified.

The data were structured and evaluated according to predetermined standards, including author, published year, country, study type, sample size, participant characteristics, intervention characteristics, comparators, outcome measures, time points, estimated effect, and outcomes. The final decision was made based on the consensus of both researchers (SXX & QL). When outcome data were unclear, we contacted the study advisor for clarification.

### Risk of bias assessment

The research dyad (SXX & QL) independently evaluated the included studies’ risk of bias using the Cochrane risk-of-bias (ROB) version 2 tool for randomized trials [23] across five domains: selection bias, performance bias, attrition bias, reporting bias, and other biases. They rated the risk at three levels: low, some concerns, and high. When the two researchers initially disagreed, they discussed the issue to reach a consensus. If no agreement could be reached, a third reviewer was consulted to resolve the discrepancy.

### Data analysis

We conducted a statistical meta-analysis focusing on disease status by extracting and analyzing outcome measures at the 3-month time point of the telehealth interventions. Subgroup analyses were performed using dialysis modality (HD or PD) and intervention methods.

We used mean differences (MD) with 95% confidence intervals (CIs) for continuous outcomes. Before pooling, outcome units were harmonized across trials using standard conversion factors to ensure comparability of mean differences. When feasible, missing standard deviations (SDs) were imputed from standard errors [24]. All meta-analyses were conducted using Review Manager (RevMan) Web (Cochrane Collaboration).

Because telehealth research often involves diverse study designs, we used random-effects models to account for potential variation in the true effect size across studies. We assessed statistical heterogeneity using the I^2^ statistic. In cases of substantial heterogeneity (I^2^ ≥ 75%), we first conducted subgroup analyses to identify potential sources of heterogeneity. If heterogeneity remained unexplained or the data were insufficient, we provided a narrative synthesis instead of a pooled estimate. Finally, we used forest plots (generated via RevMan Web) to visually represent the individual and pooled estimates for each outcome examined.

### Quality assessment

The quality of evidence across studies was assessed by applying the Grading of Recommendations, Assessment, Development, and Evaluation (GRADE) approach [25]. Evidence quality was assessed regarding the risk of bias, inconsistency, indirectness, imprecision, and publication bias of the studies for each outcome using the GRADE profiler (GRADEpro), and overall quality for each outcome (quality of the body of evidence) was graded (high, medium, low, and very low). Subgroup analyses were considered exploratory. When fewer than three studies were available for a subgroup, results were interpreted with substantial caution. The results are presented in the summary of findings table.

## Results

### Study selection

**Fig. 1:**
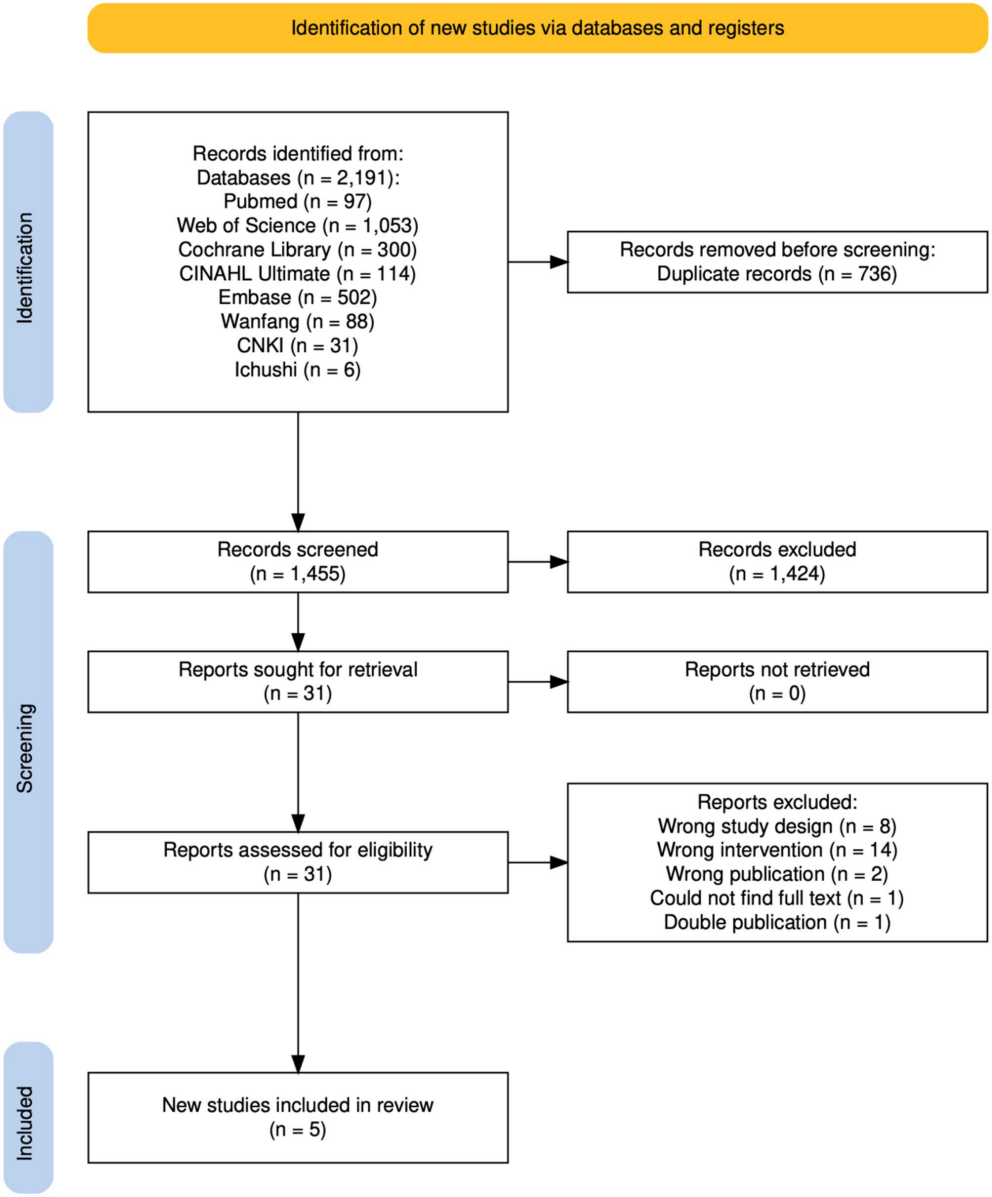
Preferred reporting items for systematic reviews and meta-analyses (PRISMA) flow diagram for trial selection

The literature search identified 2191 articles, and after excluding duplicates, 1455 were subjected to primary screening. This resulted in 31 articles that underwent secondary screening. Applying the inclusion and exclusion criteria to these led to the final acceptance of five articles (Fig. 1). Other studies (*n* = 25) were excluded (Fig. 1).

### Characteristics of the reviewed articles

Table 1 summarizes the characteristics of the included studies. All five studies were RCTs published between 2019 and 2024. Participants were from China (*n* = 2), Thailand (*n* = 2), and Iran (*n* = 1). Participants were patients on HD (2 studies) and PD (3 studies). The comparison groups typically received usual or routine care. The sample sizes ranged from 54 to 136 participants. The interventions mainly involved tele-education combined with telemonitoring [26–28], and tele-education alone.

**Table 1:** Characteristics of the reviewed articles

First author, Year of publication	Country of origin	Study design, no. of arms	Dialysis Methods	Participant characteristics	Intervention period (month), Frequency of intervention	Intervention contents	ICT/technology	Outcome Clinical Indicator	Measurement time of outcome indicators
Pungchompoo et al. 2024 [27]	Thailand	Parallel-group RCT, 2 arms	HD	*n* = 54 Intervention group: *n* = 24; Mean age 71.7 years; M/F 37.5/62.5%	6 months, continuous monitoring	**Intervention group:** Telehealth interventions: Education + Monitoring Telemonitoring: Participants were provided with a tablet and a web-based application for health monitoring. They recorded data such as body weight and health symptoms, scheduled appointments, and communicated with nurses through video calls and group chats. Data from the app were automatically transmitted to a secure server, allowing healthcare providers to track patient progress and intervene as necessary. The system flagged abnormalities in patient data, triggering follow-up actions by healthcare providers. Healthcare Providers Delivering the Interventions: By a multidisciplinary team, including renal nurse specialists and nephrologists.	iPad app + web application with user/admin panels; video visits; messaging (e.g., Facebook/Line)	SCr, Hb, Hct, Alb	Baseline, 3 and 6 months
				Control group: *n* = 30; Mean age 71.6 years; M/F 60.0/40.0%		**Control group:** Standard care included comprising routine laboratory and physical examinations, nurse-delivered health education and counselling, and a scheduled monthly clinic follow-up.			
Arad et al. 2021 [29]	Iran	Parallel-group RCT, 2 arms	HD	*n* = 66 Intervention group: *n* = 33; mean age 27.0 years; M/F 54.4/45.5%	3 months, weekly follow-ups via telephone	**Intervention group:** Telehealth interventions: Education- only Educational Support: Nurse-led telephone follow-ups were conducted twice weekly, with each call lasting approximately 20 minutes. Nurses provided guidance on treatment adherence and addressed patient concerns. Healthcare Providers Delivering the Interventions: Nurses conducted all educational sessions.	Telephone + daily SMS (patient education on diet, meds, fluids)	SCr, Hb, Alb	Baseline, 3 months
				Control group: *n* = 33; Mean age 30.0 years; M/F 57.6/42.4%		**Control group:** Standard care included the usual in-unit dialysis sessions with staff available to answer questions during or after treatment, and no additional educational or follow-up intervention.			
Sriyuktasuth et al. 2023 [28]	Thailand	Parallel-group RCT, 2 arms	PD	*n* = 104 Intervention group: *n* = 52; mean age 52.8 years; M/F 59.6/40.4%	6 months, daily telehealth intervention	**Intervention group:** Telehealth interventions: Education + Monitoring Educational Support: The application provided educational resources such as video clips and text materials Telemonitoring: The intervention utilized a mobile application for patients and a web-based system for healthcare providers. Patients recorded daily dialysis-related data (e.g., fluid exchange, body weight) and uploaded photographs of exit sites and dialysis fluids for review. The healthcare team regularly monitored this data through a secure web interface and flagged alerts for values exceeding predefined thresholds. Healthcare Providers Delivering the Interventions: The healthcare team, including PD nurses, dieticians, and technical officers, provided ongoing support reviewed patient-submitted data, and coordinated care as needed.	Patient-side mobile app + dialysis-center web app (PD Easy) linked to central server; alerts when values exceed thresholds; not for emergencies	Hct, Alb	Baseline, after 3 months, 6 months
				Control group: *n* = 52; Mean age 51.7 years; M/F 55.8/44.2%		**Control group:** Standard care			
Xu, 2023 [30]	China	Parallel-group RCT, 2 arms	PD	*n* = 60 Intervention group: *n* = 30; Mean age - years; M/F - (not reported)/- (not reported)%	M6 months, online sessions three times a week (Monday, Wednesday, Friday)	**Intervention group:** Telehealth interventions: Education-only Educational Support: Patients were educated through multimedia formats, including weekly health education delivered via WeChat or QQ groups (Mondays, Wednesdays, Fridays). Healthcare Providers Delivering the Interventions: A dedicated health management team consisting of PD physicians, nurses, nutritionists, psychologists, and engineers was formed.	WeChat/QQ groups; nightly nurse-patient check-ins; multimedia teaching; individualized plans	SCr,Hb, Alb	Baseline, after 3 months, 6 months
				Control group: *n* = 30; Mean age - years; M/F - (not reported)/- (not reported)%		**Control group:** Standard care included peritoneal-dialysis treatment with usual nursing that included health education, aseptic-technique instruction and psychological support, but no Internet-based management.			
Liao et al. 2019 [26]	China	Parallel-group RCT, 2 arms	PD	*n* = 136 Intervention group: *n* = 68; Mean age - years; M/F - (not reported)/- (not reported)%	3 months, with continuous online management	**Intervention group:** Telehealth interventions: Education + Monitoring Educational Support: The team coordinated regular online follow-ups via WeChat or QQ. Telemonitoring: Patients were provided access to a health management platform that used cloud computing and big data technologies. They uploaded health indicators such as blood pressure, weight, and biochemical data. A server-side system monitored these inputs, flagged abnormal values, and notified healthcare providers for timely interventions. Patients received automatic warnings and follow-up consultations when data exceeded safe thresholds. Healthcare Providers Delivering the Interventions: A professional health management team of 12 experts, including nephrologists, specialized nurses, nutritionists, and psychologists, was assembled.	Hospital cloud platform; patient smart-terminal (smartphone/PC) for uploads, indicator monitoring, online consult; team manages via server backend	Alb, SCr, Hb	Baseline, after 1 month, 3 months
				Control group: *n* = 68 Mean age - years; M/F - (not reported)/- (not reported)%		**Control group:** Standard care included guidance on diet and fluid restriction, complication management, monitoring of blood pressure, weight, urine volume and ultrafiltration, technical instruction on catheter exit-site care and exchanges, medication and nutrition education, exercise planning, psychological support, aseptic technique and hand-hygiene training, home-environment management and disease-prevention counselling, together with scheduled telephone or home follow-ups.			

*Note.* RCT = Randomized Controlled Trial; *M* = male; F = female; Hb = Hemoglobin; Hct = Hematocrit; Alb = Albumin; SCr = Serum Creatinine; CKD = Chronic Kidney Disease; PD = Peritoneal Dialysis; HD = Hemodialysis

### Characteristics of telehealth interventions

The interventions included in this review spanned three main approaches—tele-education plus monitoring, monitoring-only, and education-only—each varying in frequency, duration, and intensity. Pungchompoo et al. [27] implemented six months of continuous telemonitoring via a tablet- and web-based application, supplemented by scheduled educational sessions. Sriyuktasuth et al. [28] used daily telehealth contacts via an app to capture dialysis-related data and deliver regular educational support. Liao et al. [26] introduced a three-month online platform that enabled patients to upload physiological indicators and receive dietary and treatment advice through group chats.

In contrast, two studies delivered education-only interventions with minimal or no structured data monitoring [29, 30]. Arad et al. [29] conducted three-month follow-up calls twice weekly, emphasizing nurse-led educational guidance on dialysis adherence and diet. Xu et al. [30] provided thrice-weekly health education for six months via multimedia platforms but did not require ongoing biometric submissions.

### Risk of bias

**Fig. 2:**
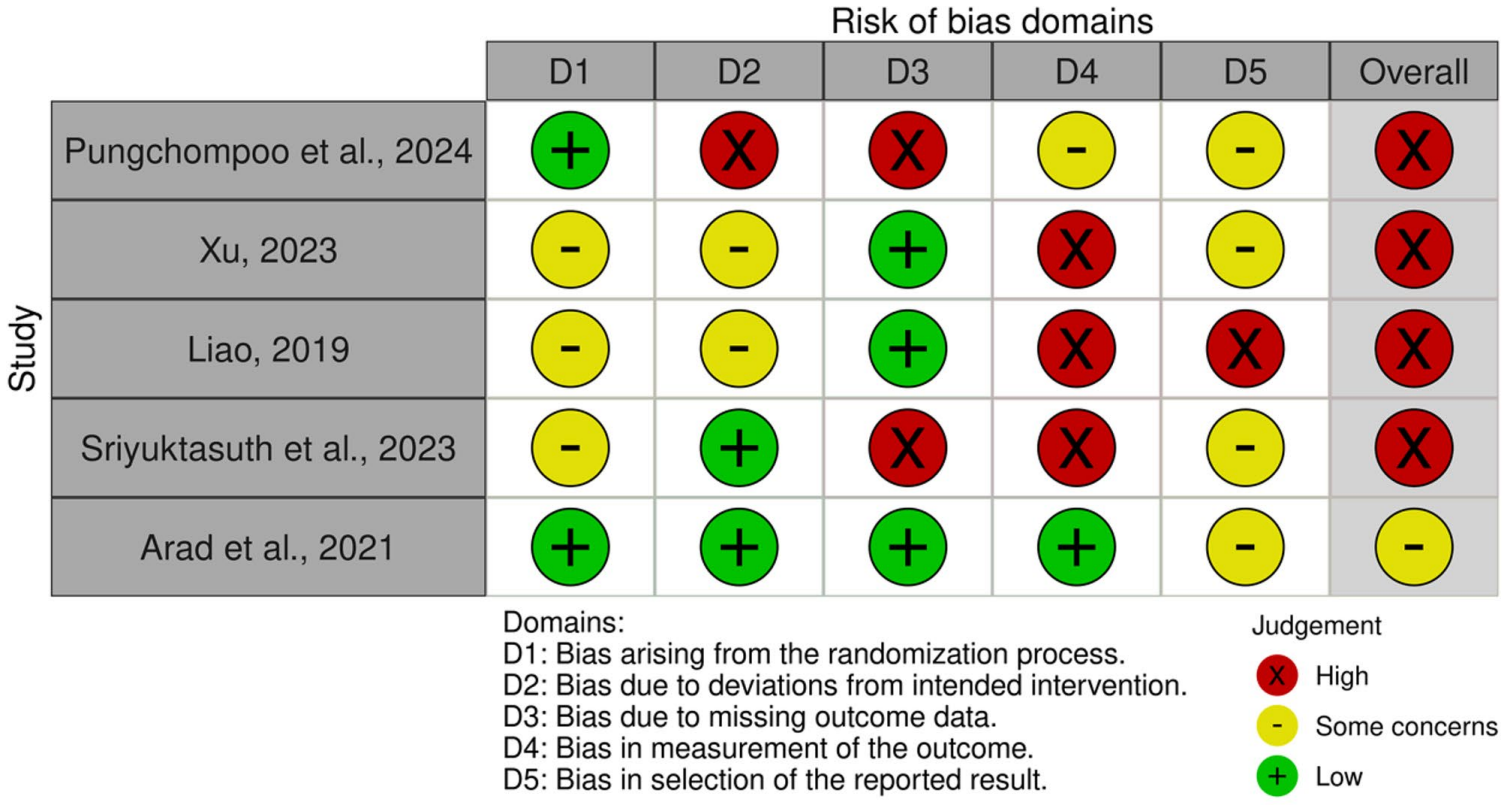
Risk of bias assessment according the Cochrane Risk-of-bias 2.0 Tool＊- review authors’ domain-level judgements for each included study

Among the five included RCT studies, two provided detailed descriptions of how the random sequence was generated [26, 27]. However, four studies did not specify how allocation concealment was implemented, resulting in an unclear risk of selection bias. Due to the nature of the interventions, none of the studies could blind participants and personnel, leading to a high risk of performance bias. Two studies [26, 29] mitigated detection bias by blinding outcome assessors. One study [27] reported baseline imbalances between treatment groups, including differences in income levels, dialysis duration, and comorbidities. Additionally, only one study explicitly reported using an intention-to-treat (ITT) analysis [28]. Two studies [26, 29] reported a dropout rate of less than 5%, whereas the remaining studies had higher dropout rates, thereby posing a higher risk of statistical bias. Two studies [28, 29] provided research protocols; however, once of the protocols [28] did not include details of the analysis methods employed. A summary of the risk of bias assessment is presented in Fig. 2, and all outcomes are synthesized qualitatively or quantitatively in Table 2.

**Table 2:** Interdisciplinary telehealth studies and reported outcomes

Clinical outcomes	Number of studies	Total number of participants	References
Albumin	5	420	Arad et al. 2021 [29]
			Pungchompoo et al. 2024 [27]
			Sriyuktasuth et al. 2023 [28]
			Xu et al. 2023 [30]
			Liao et al. 2019 [26]
Serum Creatinine	4	316	Liao et al. 2019 [26]
			Pungchompoo et al. 2024 [27]
			Arad et al. 2021 [29]
			Xu et al. 2023 [30]
Hemoglobin	4	316	Liao et al. 2019 [26]
			Pungchompoo et al. 2024 [27]
			Arad et al. 2021 [29]
			Xu et al. 2023 [30]
Hematocrit	2	158	Pungchompoo et al. 2024 [27]
			Sriyuktasuth et al. 2023 [28]

### Comparison of telehealth program by adherence level

A meta-analysis compared interdisciplinary telehealth with usual care for each outcome (Table 3). Because heterogeneity was substantial for the overall albumin (I^2^ = 98%) and serum creatinine (I^2^ = 85%) analyses, we did not pool an overall effect estimate for these outcomes. Pooled results indicated that telehealth was associated with higher hemoglobin levels at approximately 3 months (MD = 8.40; 95% CI [5.82, 10.99]; I^2^ = 0%; *p* < 0.00001; total *N* = 155/161). For hematocrit, the pooled estimate did not differ significantly between groups (MD = 1.04; 95% CI [−0.82, 2.91]; I^2^ = 0%; *p* = 0.27; total *N* = 76/82).

**Table 3:** Results of meta-analysis

Outcomes	Participants (IG/CG)	Effect measures
Albumin	207/213	Not pooled due to very high heterogeneity (I^2^ = 98%)
Serum Creatinine	155/161	Not pooled due to very high heterogeneity (I^2^ = 85%)
Hemoglobin	155/161	MD = 8.40; 95% CI = [5.82, 10.99]; *p* < 0.001; I^2^ = 0%
Hematocrit	76/82	MD = 1.04; 95% CI = [−0.82, 2.91]; *p* = 0.27; I^2^ = 0%

*Note*: Abbreviations: CG, control group; CI, confidence interval; IG, intervention group; MD, Mean difference

### Comparison of biomarkers between PD and HD

#### Albumin

We synthesized data from five studies [26–30] stratified by dialysis modality into HD [27, 29] and PD groups [26, 28, 30]. High heterogeneity was found in both HD (I^2^ = 86%) and PD groups (I^2^ = 99%). Neither subgroup achieved statistically significant results. Therefore, we were unable to synthesize the data.

#### Serum creatinine

**Fig. 3:**
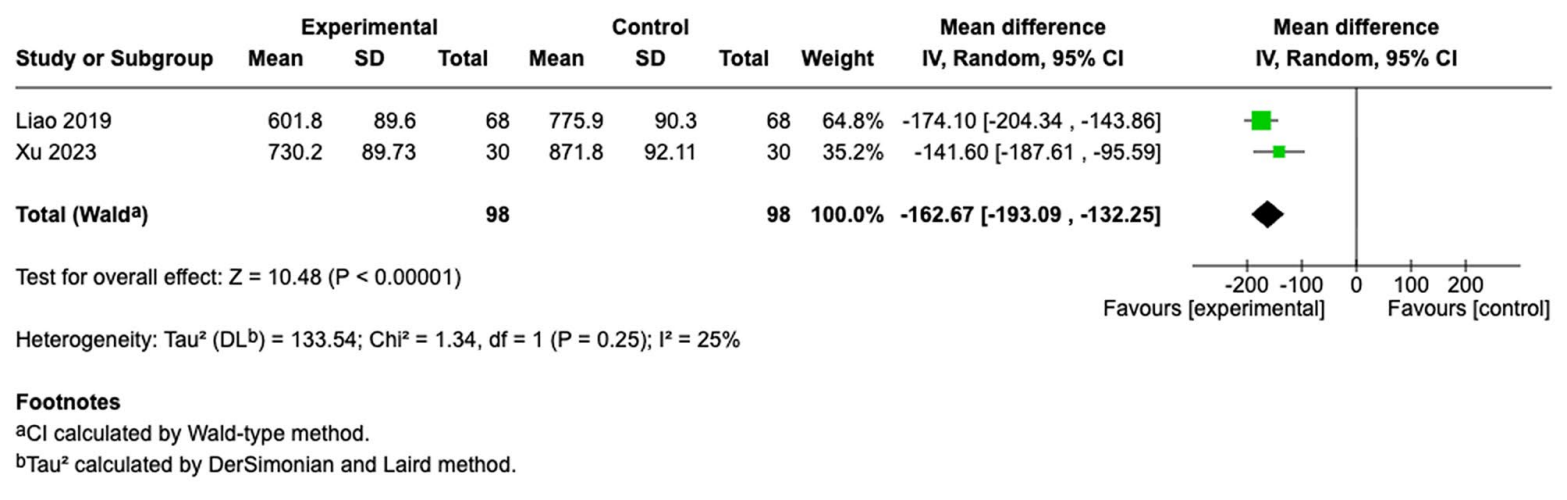
Forest plot of serum creatinine comparison of telehealth vs. usual care by PD modality

**Fig. 4:**
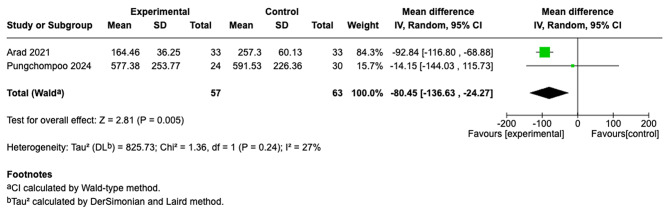
Forest plot of serum creatinine comparison of telehealth vs. usual care by HD modality

We summarized findings from four studies [26, 27, 29, 30]. In the PD group [26, 30], the results showed that the intervention group was statistically significant (MD = −162.67; 95% CI [−193.09, −132.25]; I^2^ = 25%; *p* < 0.00001) (Fig. 3). Similarly, in the HD group, which showed reduced serum creatinine levels [27, 29], the results were also statistically significant for reducing serum creatinine levels (MD = −80.45; 95% CI [−136.63, −24.27]; I^2^ = 27%; *p* = 0.005). However, because each subgroup included only two studies, these subgroup estimates and the test for subgroup differences should be considered exploratory and interpreted with substantial caution (Fig. 4).

#### Hemoglobin

**Fig. 5:**
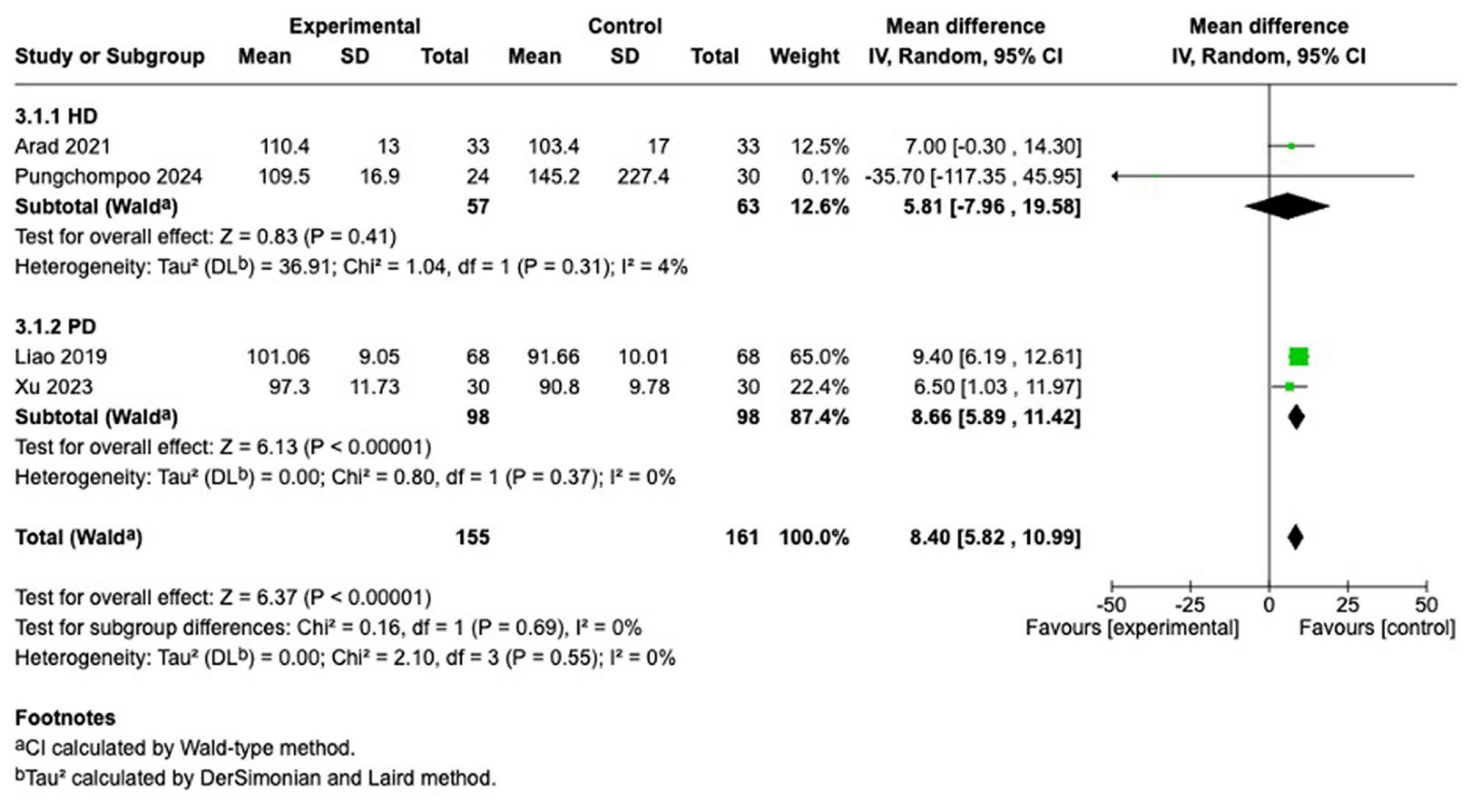
Forest plot of hemoglobin comparison of telehealth vs. usual care, with subgroup analyses by dialysis modality

We synthesized data from four studies [26, 27, 29, 30]. Significant improvement in hemoglobin levels was observed in PD group [26, 30] (MD = 8.66 [5.89, 11.42], *p* < 0.00001, I^2^ = 0%). In contrast, HD patients [27, 29] showed no statistically significant improvement. Subgroup difference (Chi^2^ = [0.16], df = 1, *p* = [0.69]). Given that each subgroup was based on only two studies, these findings are hypothesis-generating and should be interpreted cautiously (Fig. 5).

#### Hematocrit

Two studies evaluated hematocrit levels [27, 28], but one focused on PD and the other on HD. Therefore, a meta-analysis could not be performed within this subgroup.

### Comparison among different telehealth intervention models

#### Albumin

Subgroup analyses compared education combined with monitoring [26–28] versus education-only interventions [29, 30]. The education-only group did not reach statistical significance, whereas the education combined with monitoring group showed high heterogeneity (I^2^ = 75%).

#### Serum creatinine

In the education combined with monitoring group [26, 27] and education-only group [29, 30], high heterogeneity persisted in both the education combined with monitoring group (I^2^ = 82%) and education-only group (I^2^ = 71%) subgroups.

#### Hemoglobin

**Fig. 6:**
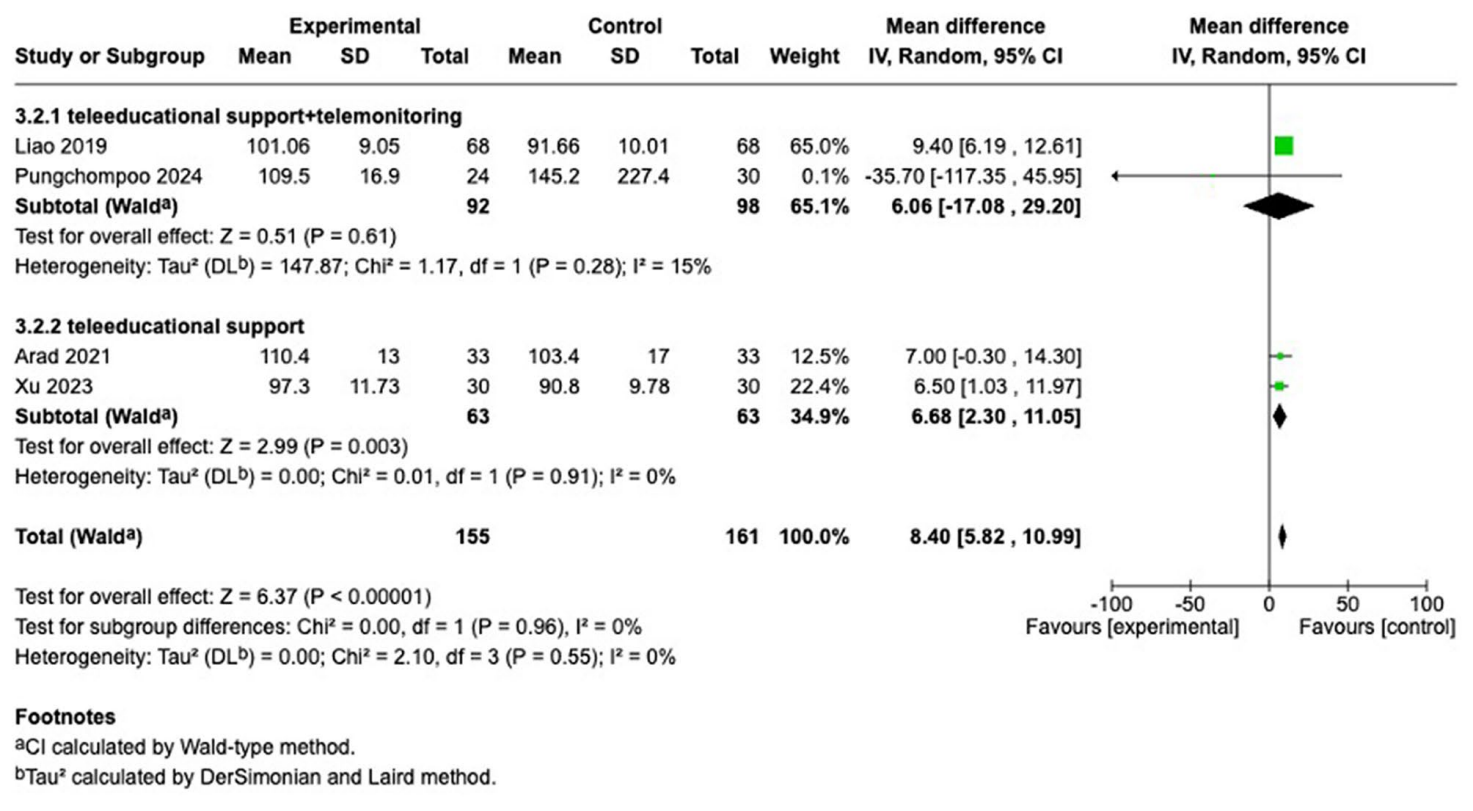
Forest plot comparing telehealth modality usual care on hemoglobin

Further subgroup analyses based on intervention type revealed that the education combined with the monitoring group [26, 27] did not reach statistical significance. However, the education-only group [29, 30] exhibited low heterogeneity, with statistically significant results (MD = 6.68; 95% CI [2.30, 11.05]; I^2^ = 0%; *p* = 0.003). However, because each subgroup included only two studies, these subgroup estimates and the test for subgroup differences (Chi^2^ = [0.00], df = 1, *p* = [0.96]) should be considered exploratory and interpreted with substantial caution. (Fig. 6).

#### Hematocrit

**Fig. 7:**
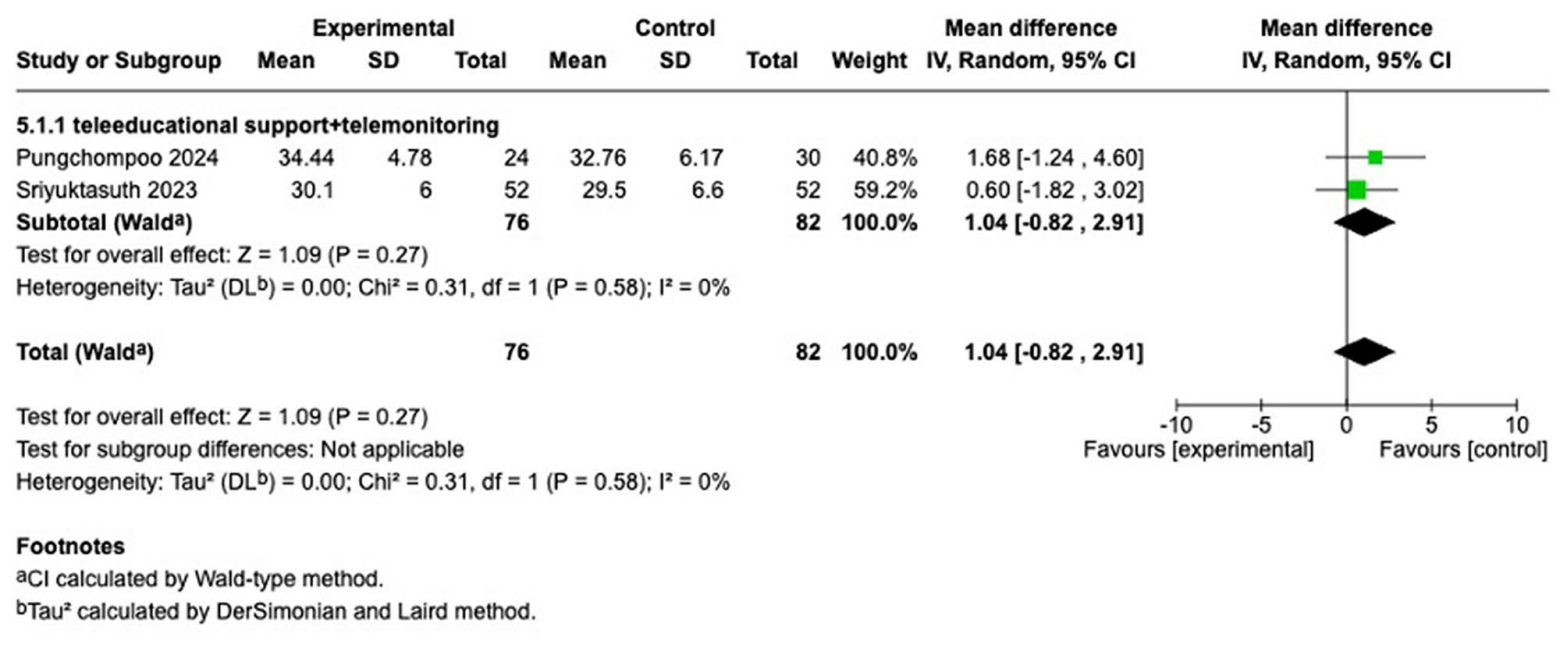
Forest plot comparing telehealth modality usual care on hematocrit

Two studies [27, 28] were synthesized. Both studies belong to the education combined with the monitoring group. The analysis revealed no statistically significant differences between the intervention and control groups (MD = 1.04; 95% CI [−0.82, 2.91]; I^2^ = 0%; *p* = 0.27), Given that each subgroup was based on only two studies, these findings are hypothesis-generating and should be interpreted cautiously (Fig. 7).

### Summary of findings

We assessed the certainty of evidence for the prespecified clinical indicators. Evidence certainty was low for serum creatinine at 3 months in hemodialysis patients and for hemoglobin at 3 months in the tele-educational-support subgroup. In contrast, certainty was very low for both serum creatinine and hemoglobin at 3 months in peritoneal-dialysis patients. All four outcomes were downgraded because the contributing trials had a high overall risk of bias and small total sample sizes, leading to imprecision. Details are presented in Table 4.

**Table 4:** Summary of findings

Telehealth interventions by nurses and other healthcare providers compared to usual care for adults undergoing maintenance dialysis			
Patient/Population: Adults receiving maintenance dialysis Setting: Home/outpatient care Intervention: Telehealth interventions involving interactive communication between healthcare professionals and patients using ICT. These interventions may include providing educational support, monitoring physical and mental health status, and verifying health-related information. Comparison: Usual care			
Outcomes	Anticipated absolute effects* (95% CI)	No. of participants (studies)	Certainty of the evidence (GRADE)
	Risk with Main Comparison		
Serum Creatinine 3 months HD	MD 80.45 lower (136.63 lower to 24.27 lower)	120 (2 RCTs)	⨁⨁◯◯ Low^b,c^
Serum Creatinine 3 months PD	MD 162.67 lower (193.09 lower to 132.25 lower)	196 (2 RCTs)	⨁◯◯◯ Very low^a,b^
Hemoglobin 3 months PD	MD 8.66 higher (5.89 higher to 11.42 higher)	196 (2 RCTs)	⨁◯◯◯ Very low^a,b^
Hemoglobin 3 months only Tele Educational Support	MD 6.68 higher (2.3 higher to 11.05 higher)	126 (2 RCTs)	⨁⨁◯◯ Low^b,c^

*Notes:* *The risk in the intervention group (and its 95% confidence interval) is based on the assumed risk in the comparison group and the relative effect of the intervention (and its 95% CI)

CI: confidence interval; MD: mean difference; RR: risk ratio; SMD: standardized mean difference; PD: peritoneal dialysis; HD: hemodialysis

GRADE Working Group grades of evidence

High certainty: we are very confident that the true effect lies close to that of the estimate of the effect

Moderate certainty: we are moderately confident in the effect estimate: the true effect is likely to be close to the estimate of the effect, but there is a possibility that it is substantially different

Low certainty: our confidence in the effect estimate is limited: the true effect may be substantially different from the estimate of the effect

Very low certainty: we have very little confidence in the effect estimate: the true effect is likely to be substantially different from the estimate of effect

a. Two level downgraded for that the pooled effect provided by studies with high risk of bias in overall

b. Too small a sample size may lead to imprecise estimates

c. One level downgraded for that the pooled effect provided by studies with high risk of bias in overall

## Discussion

### Characteristics and quality of RCTs

Methodologically, the five included RCTs presented notable gaps in reporting. Two studies did not provide any baseline demographic or clinical data, which makes it challenging to confirm group comparability and thus complicates result interpretation [31]. While three studies mentioned random sequence generation, allocation concealment remained unclear in the majority. Moreover, no study comprehensively assessed blinding of participants and personnel, thereby increasing the risk of performance bias. One study [27] reported baseline imbalances in income levels, yet the authors did not specify how they accounted for these differences in their analysis. Only one trial [28] explicitly stated that it used an intention-to-treat approach, leaving it unclear whether dropouts were handled consistently in the other studies. These limitations collectively raise concerns about internal validity. Although dropout rates varied-three studies reported less than 5%. Those with higher attrition had incomplete or unclear descriptions of how missing data were managed, potentially inflating the risk of bias. The small sample sizes in many included trials may limit the generalizability of the findings and increase the risk of statistical bias, further compounded by the high heterogeneity of outcome measures. Consequently, the GRADE assessment often indicated low or very low certainty across outcomes, underscoring the methodological fragility of the available evidence.

### Summary of participants

Participant profiles differed markedly across the five studies, particularly regarding age ranges and dialysis modality. In Pungchompoo et al. [27] from Thailand, older adults (mean age > 65) formed the majority, posing potential challenges with telehealth adoption—mainly if digital literacy or caregiver support was limited. By contrast, Arad et al. [29] reported a mean age of only 27, indicating a younger cohort who may adapt more readily to the app- or phone-based interventions. This stark age difference could partly explain the substantial heterogeneity observed when pooling these studies, as older adults often require additional time and tailored support to master telehealth technologies [32]. Additionally, despite these distinctions, few studies thoroughly stratified outcomes by age, gender, or comorbidities, making it challenging to determine which subgroups derive the most significant benefit from each telehealth model.

### Summary of evidence

Our findings suggest that telehealth interventions can lead to various positive short-term effects on key clinical indicators in patients undergoing maintenance dialysis. Combined analyses showed a significant decline in serum creatinine levels for both HD and PD patients. Importantly, in maintenance dialysis, serum creatinine mainly reflects the balance between its production and removal and is heavily influenced by muscle mass, nutrition, and dilution; it does not indicate kidney recovery. Therefore, any observed reduction should be interpreted carefully and alongside other measures such as adequacy indices such as Kt/V, nutritional markers (albumin, nPCR), and the overall clinical picture. In this review, we consider creatinine a supportive surrogate marker rather than a standalone indicator of clinical improvement. Regarding anemia management, PD patients showed a more notable improvement in hemoglobin levels. This suggests that, compared with HD patients—who require frequent hospital visits—those on PD—who typically receive treatment at home—have fewer follow-up sessions [33]. Patients on PD are more likely to benefit from timely, convenient telehealth guidance to further improve clinical outcomes.

However, high heterogeneity (I^2^) values were common, likely caused by multiple factors. First, interventions ranged from continuous monitoring to minimal oversight, reducing the reliability of pooled estimates. Second, baseline differences—especially in age, comorbidities, and socioeconomic status—were not precisely controlled for. Third, specific outcomes, such as hematocrit, were reported in only 2 small trials, making the analyses underpowered and less generalizable. Additional methodological weaknesses, such as incomplete allocation concealment, inconsistent handling of missing data, and non-standardized outcome measurements—including different local laboratory methods, unit conversions, and imputed standard deviations—further increased random error across studies.

### Differences between HD and PD patients using telehealth

Regarding serum creatinine and hemoglobin, PD patients who perform dialysis at home generally benefit from individualized guidance through remote education and monitoring. This advantage was evidenced by their marked improvement in hemoglobin levels, consistent with prior findings that continuous support outside conventional clinic visits can enhance clinical outcomes in PD [11]. HD patients, on the other hand, must regularly travel to dialysis centers, where much of their care is delivered in person by healthcare professionals; consequently, telehealth might offer a smaller incremental benefit in this setting [34].

Furthermore, PD patients in some areas of China and Thailand face logistical and resource-related barriers, including transportation challenges and limited healthcare facilities [35, 36]. In Iran, one major reason for the low adoption rate of peritoneal dialysis is insufficient patient health literacy and limited access to reliable information about PD [37]. Telehealth can help mitigate these issues by providing timely professional support. For instance, Xu et al. [30] reported that telehealth-guided interventions among rural older adults receiving PD led to significantly improved clinical outcomes. Additionally, telehealth can enhance PD patients’ health literacy by providing accessible, continuous education [38].

### Study limitations

This review has several limitations. First, the language restriction—limiting searches to English, Chinese, and Japanese—may have excluded relevant studies published in other languages, introducing a risk of language bias. Second, by focusing only on RCTs, many subgroup analyses were based on only two trials per subgroup; therefore, subgroup findings are exploratory and highly uncertain, and should not be used to draw firm conclusions about effect modification or model superiority. We excluded observational and qualitative studies that could provide additional insights into patient adherence, satisfaction, or practical barriers to telehealth implementation. Third, our search strategy was guided by a predefined set of terms and databases, which might have overlooked gray literature or ongoing clinical trials. Fourth, most studies were conducted in China and Southeast Asia, with small sample sizes and few multi-center or cross-national trials. This geographic concentration limits the external validity of the results for other populations and healthcare systems. Several trials did not report basic participant characteristics such as age and sex, limiting the assessment of baseline comparability and reducing confidence in the applicability of findings. Most trials were open-label and lacked allocation concealment, increasing the risk of performance and detection bias. While our primary endpoints were laboratory measures—reducing observer-assessment bias—adherence-related effects and co-interventions could still influence outcomes. These factors, along with small sample sizes, should temper any conclusions about effectiveness. We also recognize the absence of patient-centered endpoints, such as quality of life, adherence, and hospitalization, in our analyses. Due to heterogeneous instruments and limited early time-point data, we did not include these outcomes here; we plan to address them explicitly in future research.

### Suggestions for further research

Future inquiries might focus on three key areas. First, large-scale, multicenter randomized controlled trials across diverse health systems with longer follow-up—ideally using hybrid effectiveness–implementation designs—could provide more accurate estimates of long-term effectiveness and sustainability while clarifying staffing, workflow, and digital infrastructure needs for scalable telehealth in dialysis. Second, rigorous cost-effectiveness evaluations are necessary to understand the financial implications of wider telehealth adoption and to guide resource allocation without sacrificing quality of care. Third, outcome measurement should go beyond biochemical markers to include standardized, patient-centered endpoints assessed at common time points, such as quality of life, adherence, hospitalization, patient satisfaction, and psychological well-being.

## Conclusions

This systematic review and meta-analysis suggests that telehealth interventions may be associated with short-term changes in several laboratory indicators among patients receiving maintenance dialysis, particularly those on peritoneal dialysis. Evidence of benefit was more consistent for anemia-related management such as hemoglobin in PD subgroups, whereas findings for nutritional markers such as albumin were inconsistent. Although reductions in serum creatinine were observed in some analyses, serum creatinine in maintenance dialysis is influenced by multiple factors; therefore, this finding should be interpreted cautiously. Across the limited available trials, effects varied by intervention intensity and study context; however, due to the small number of studies within each intervention model, no firm conclusions can be drawn regarding the superiority of any single telehealth model.

The small sample sizes, short intervention durations, and limited geographic scope of the included studies restrict the generalizability of these findings and increase the risk of bias. Additionally, high heterogeneity across interventions and outcome measures highlights the need for standardized protocols. Moving forward, larger multi-center RCTs with longer follow-ups, along with cost-effectiveness assessments and broader outcome measures such as patient satisfaction and quality of life, are crucial for determining the best design and implementation of telehealth services for dialysis care.

## Electronic supplementary material

Below is the link to the electronic supplementary material.

Supplementary Material 1

## Data Availability

All data generated or analysed during this study are included in this published article and its supplementary information files.
