# Supplementary material for: Short-term effectiveness of telehealth interventions for people in maintenance dialysis: differential impacts on clinical outcomes by modality and intervention type - a systematic review and meta-analysis
Source: BMC Nephrol. 2026 Feb 20;27:195. doi: 10.1186/s12882-026-04833-0 (PMC13032427; doi:10.1186/s12882-026-04833-0)
Supplement: Supplementary file 1 — Supplementary Material 1 [file 12882_2026_4833_MOESM1_ESM.docx]

Appendix S1

*Searched Databases and Keyword*

| Pubmed, until 26 July 2024 | |
| --- | --- |
| #1 | (("telehealth"[Title/Abstract] OR "Telemedicine"[Title/Abstract] OR "telemedical"[Title/Abstract]) AND "Telenursing"[Title/Abstract]) OR "telemonitoring"[Title/Abstract] OR "Telerehabilitation"[Title/Abstract] OR "telepractice"[Title/Abstract] OR "telecare"[Title/Abstract] OR "e-health"[Title/Abstract] OR "digital health"[Title/Abstract] OR "mobile health"[Title/Abstract] OR "digital interventions"[Title/Abstract] OR "Telemedicine"[MeSH Terms] OR "Telenursing"[MeSH Terms] OR "Telerehabilitation"[MeSH Terms] |
| #2 | "kidneys artificial"[Title/Abstract] OR "renal insufficiency"[Title/Abstract] OR (("Peritoneal Dialysis"[MeSH Terms] OR ("Peritoneal"[All Fields] AND "Dialysis"[All Fields]) OR "Peritoneal Dialysis"[All Fields]) AND "continuous ambulatory"[Title/Abstract]) OR "Peritoneal Dialysis"[Title/Abstract] OR "Continuous Renal Replacement Therapy"[Title/Abstract] OR (("haemodialysis"[All Fields] OR "Renal Dialysis"[MeSH Terms] OR ("Renal"[All Fields] AND "Dialysis"[All Fields]) OR "Renal Dialysis"[All Fields] OR "Hemodialysis"[All Fields]) AND "units hospital"[Title/Abstract]) OR "hemodialysis home"[Title/Abstract] OR "Renal Dialysis"[Title/Abstract] OR "Kidney Diseases"[Title/Abstract] OR "Chronic Kidney Diseases of Uncertain Etiology"[Title/Abstract] OR ("chronic kidney disease mineral"[Title/Abstract] AND "bone disorder"[Title/Abstract]) OR "renal insufficiency chronic"[Title/Abstract] OR "Kidney Transplantation"[MeSH Terms] OR "peritoneal dialysis, continuous ambulatory"[MeSH Terms] OR "Peritoneal Dialysis"[MeSH Terms] OR "Continuous Renal Replacement Therapy"[MeSH Terms] OR "hemodialysis units, hospital"[MeSH Terms] OR "hemodialysis, home"[MeSH Terms] OR "Renal Dialysis"[MeSH Terms] OR "Kidney Diseases"[MeSH Terms] OR "Chronic Kidney Diseases of Uncertain Etiology"[MeSH Terms] OR "Chronic Kidney Disease-Mineral and Bone Disorder"[MeSH Terms] OR "renal insufficiency, chronic"[MeSH Terms] OR "kidneys, artificial"[MeSH Terms] |
| #3 | ("randomized controlled trial"[Publication Type] OR "controlled clinical trial"[Publication Type] OR "randomized"[Title/Abstract] OR "placebo"[Title/Abstract] OR "clinical trials as topic"[MeSH Terms:noexp] OR "randomly"[Title/Abstract] OR "trial"[Title]) NOT ("animals"[MeSH Terms] NOT "humans"[MeSH Terms]) |
| #4 | #1AND #2AND#3 |
| Cochrane Library, until 26 July 2024 | |
| #1 | (telehealth):ti,ab,kw OR (telemedicine):ti,ab,kw OR (telemedical):ti,ab,kw OR (telenursing):ti,ab,kw OR (telemonitoring):ti,ab,kw OR (telerehabilitation):ti,ab,kw OR (telepractice):ti,ab,kw OR (telecare):ti,ab,kw OR (e-health):ti,ab,kw OR (digital health):ti,ab,kw OR (mobile health):ti,ab,kw OR (digital interventions):ti,ab,kw |
| #2 | MeSH descriptor: [Telemedicine] explode all trees |
| #3 | MeSH descriptor: [Telenursing] explode all trees |
| #4 | MeSH descriptor: [Telerehabilitation] explode all trees |
| #5 | #1 or #2 or #3 or #4 |
| #6 | (kidneys artificial):ti,ab,kw OR (renal insufficiency):ti,ab,kw OR (((peritoneal AND dialysis) OR (peritoneal dialysis)) AND (continuous ambulatory):ti,ab,kw) OR (peritoneal dialysis):ti,ab,kw OR (continuous renal replacement therapy):ti,ab,kw OR (((renal AND dialysis) OR (renal dialysis) OR (hemodialysis)) AND (units hospital):ti,ab,kw) OR (hemodialysis home):ti,ab,kw OR (renal dialysis):ti,ab,kw OR (kidney diseases):ti,ab,kw OR (chronic kidney diseases of uncertain etiology):ti,ab,kw OR ((chronic kidney disease mineral):ti,ab,kw AND (bone disorder):ti,ab,kw) OR (renal insufficiency chronic):ti,ab,kw |
| #7 | MeSH descriptor: [Peritoneal Dialysis, Continuous Ambulatory] explode all trees |
| #8 | MeSH descriptor: [Peritoneal Dialysis] explode all trees |
| #9 | MeSH descriptor: [Continuous Renal Replacement Therapy] explode all trees |
| #10 | MeSH descriptor: [Hemodialysis Units, Hospital] explode all trees |
| #11 | MeSH descriptor: [Renal Dialysis] explode all trees |
| #12 | MeSH descriptor: [Kidneys, Artificial] explode all trees |
| #13 | MeSH descriptor: [Renal Insufficiency, Chronic] explode all trees |
| #14 | MeSH descriptor: [Chronic Kidney Disease-Mineral and Bone Disorder] explode all trees |
| #15 | MeSH descriptor: [Renal Insufficiency] explode all trees |
| #16 | #6 or #7 or #8 or #9 or #10 or #11 or #12 or #13 or #14 or #15 |
| #17 | #5 AND #16 |
| Embase,until 26 July 2024 | |
| #1 | 'randomized controlled trial'/exp OR 'controlled clinical trial'/de OR random*:ti,ab,tt OR 'randomization'/de OR 'intermethod comparison'/de OR placebo:ti,ab,tt OR compare:ti,tt OR compared:ti,tt OR comparison:ti,tt OR ((evaluated:ab OR evaluate:ab OR evaluating:ab OR assessed:ab OR assess:ab) AND (compare:ab OR compared:ab OR comparing:ab OR comparison:ab)) OR ((open NEXT/1 label):ti,ab,tt) OR (((double OR single OR doubly OR singly) NEXT/1 (blind OR blinded OR blindly)):ti,ab,tt) OR 'double blind procedure'/de OR ((parallel NEXT/1 group*):ti,ab,tt) OR crossover:ti,ab,tt OR 'cross over':ti,ab,tt OR (((assign* OR match OR matched OR allocation) NEAR/6 (alternate OR group OR groups OR intervention OR interventions OR patient OR patients OR subject OR subjects OR participant OR participants)):ti,ab,tt) OR assigned:ti,ab,tt OR allocated:ti,ab,tt OR ((controlled NEAR/8 (study OR design OR trial)):ti,ab,tt) OR volunteer:ti,ab,tt OR volunteers:ti,ab,tt OR 'human experiment'/de OR trial:ti,tt |
| #2 | 'telemedicine'/exp OR 'telenursing'/exp OR 'telerehabilitation'/exp |
| #3 | telehealth:ab,ti OR telemedicine:ab,ti OR telemedical:ab,ti OR telenursing:ab,ti OR telemonitoring:ab,ti OR telerehabilitation:ab,ti OR telepractice:ab,ti OR telepractic:ab,ti OR telecare:ti,ab OR 'e health':ti,ab OR (digital:ab,ti AND health:ti,ab) OR (mobile:ab,ti AND health:ti,ab) OR (digital:ab,ti AND interventions:ti,ab) OR 'tele medicine':ti,ab OR 'virtual medicine':ti,ab OR 'tele-nursing':ti,ab OR 'virtual nursing':ti,ab |
| #4 | 'continuous ambulatory peritoneal dialysis'/exp OR 'peritoneal dialysis'/exp OR 'continuous renal replacement therapy'/exp OR 'hemodialysis'/exp OR 'home dialysis'/exp OR 'chronic kidney failure'/exp OR 'chronic kidney disease-mineral and bone disorder'/exp OR 'kidney failure'/exp |
| #5 | 'ambulatory continuous peritoneal dialysis':ab,ti OR 'capd':ab,ti OR 'peritoneum dialysis':ab,ti OR 'peritoneal dialysis':ab,ti OR 'crrt':ab,ti OR 'continuous renal replacement therapy':ab,ti OR 'chronic haemodialysis':ab,ti OR 'chronic hemodialysis':ab,ti OR 'chronic intermittent haemodialysis':ab,ti OR 'chronic intermittent hemodialysis':ab,ti OR 'intermittent chronic haemodialysis':ab,ti OR 'intermittent chronic hemodialysis':ab,ti OR 'intermittent haemodialysis':ab,ti OR 'intermittent hemodialysis':ab,ti OR 'renal dialysis':ab,ti OR 'hemodialysis':ab,ti OR 'haemodialysis':ab,ti OR 'hemodialysis center':ab,ti OR 'hemodialysis department':ab,ti OR 'hemodialysis unit':ab,ti OR 'haemodialysis center':ab,ti OR 'haemodialysis centre':ab,ti OR 'haemodialysis department':ab,ti OR 'haemodialysis unit':ab,ti OR 'haemodialysis, home':ab,ti OR 'hemodialysis, home':ab,ti OR 'home haemodialysis':ab,ti OR 'home hemodialysis':ab,ti OR 'home dialysis':ab,ti OR 'artificial kidney':ab,ti OR 'chronic kidney disease':ab,ti OR 'chronic renal failure':ab,ti OR 'chronic renal insufficiency':ab,ti OR 'kidney failure':ab,ti OR 'kidney diseases':ab,ti OR 'renal failure':ab,ti OR 'renal insufficiency':ab,ti OR 'chronic kidney diseases of uncertain etiology':ab,ti |
| #6 | ((random* NEXT/1 sampl* NEAR/8 ('cross section*' OR questionnaire* OR survey OR surveys OR database OR databases)):ti,ab,tt) NOT ('comparative study'/de OR 'controlled study'/de OR 'randomised controlled':ti,ab,tt OR 'randomized controlled':ti,ab,tt OR 'randomly assigned':ti,ab,tt) OR ('cross-sectional study'/de NOT ('randomized controlled trial'/exp OR 'controlled clinical trial'/de OR 'controlled study'/de OR 'randomised controlled':ti,ab,tt OR 'randomized controlled':ti,ab,tt OR 'control group':ti,ab,tt OR 'control groups':ti,ab,tt)) OR ('case control*':ti,ab,tt AND random*:ti,ab,tt NOT ('randomised controlled':ti,ab,tt OR 'randomized controlled':ti,ab,tt)) OR ('systematic review':ti,tt NOT (trial:ti,tt OR study:ti,tt)) OR (nonrandom*:ti,ab,tt NOT random*:ti,ab,tt) OR 'random field*':ti,ab,tt OR (('random cluster' NEAR/4 sampl*):ti,ab,tt) OR (review:ab AND review:it NOT trial:ti,tt) OR ('we searched':ab AND (review:ti,tt OR review:it)) OR 'update review':ab OR ((databases NEAR/5 searched):ab) OR ((rat:ti,tt OR rats:ti,tt OR mouse:ti,tt OR mice:ti,tt OR swine:ti,tt OR porcine:ti,tt OR murine:ti,tt OR sheep:ti,tt OR lambs:ti,tt OR pigs:ti,tt OR piglets:ti,tt OR rabbit:ti,tt OR rabbits:ti,tt OR cat:ti,tt OR cats:ti,tt OR dog:ti,tt OR dogs:ti,tt OR cattle:ti,tt OR bovine:ti,tt OR monkey:ti,tt OR monkeys:ti,tt OR trout:ti,tt OR marmoset*:ti,tt) AND 'animal experiment'/de) OR ('animal experiment'/de NOT ('human experiment'/de OR 'human'/de)) |
| #7 | #1 NOT #6 |
| #8 | #2 OR #3 |
| #9 | #4 OR #5 |
| #10 | #7 AND #8 AND #9 |
|  | |
| CINAHL Ultimate, until 26 July 2024 | |
| S1 | SU ((MH "Telemedicine") OR (MH "Telenursing") OR (MH "Telerehabilitation")) |
| S2 | TI ( telehealth OR telemedicine OR telemedical OR telenursing OR telemonitoring OR telerehabilitation OR telepractice OR telepractic OR telecare OR "e health" OR (digital AND health) OR (mobile AND health) OR (digital AND interventions) OR "tele medicine" OR "virtual medicine" OR "tele-nursing" OR "virtual nursing" ) OR AB ( telehealth OR telemedicine OR telemedical OR telenursing OR telemonitoring OR telerehabilitation OR telepractice OR telepractic OR telecare OR "e health" OR (digital AND health) OR (mobile AND health) OR (digital AND interventions) OR "tele medicine" OR "virtual medicine" OR "tele-nursing" OR "virtual nursing" ) |
| S3 | SU ((MH "Continuous Ambulatory Peritoneal Dialysis") OR (MH "Peritoneal Dialysis") OR (MH "Continuous Renal Replacement Therapy") OR (MH "Hemodialysis") OR (MH "Home Dialysis") OR (MH "Chronic Kidney Failure") OR (MH "Chronic Kidney Disease-Mineral and Bone Disorder") OR (MH "Kidney Failure")) |
| S4 | TI ( ("ambulatory continuous peritoneal dialysis" OR "capd" OR "peritoneum dialysis" OR "peritoneal dialysis" OR "crrt" OR "continuous renal replacement therapy" OR "chronic haemodialysis" OR "chronic hemodialysis" OR "chronic intermittent haemodialysis" OR "chronic intermittent hemodialysis" OR "intermittent chronic haemodialysis" OR "intermittent chronic hemodialysis" OR "intermittent haemodialysis" OR "intermittent hemodialysis" OR "renal dialysis" OR "hemodialysis" OR "haemodialysis" OR "hemodialysis center" OR "hemodialysis department" OR "hemodialysis unit" OR "haemodialysis center" OR "haemodialysis centre" OR "haemodialysis department" OR "haemodialysis unit" OR "haemodialysis, home" OR "hemodialysis, home" OR "home haemodialysis" OR "home hemodialysis" OR "home dialysis" OR "artificial kidney" OR "chronic kidney disease" OR "chronic renal failure" OR "chronic renal insufficiency" OR "kidney failure" OR "kidney diseases" OR "renal failure" OR "renal insufficiency" OR "chronic kidney diseases of uncertain etiology") ) OR AB ( ("ambulatory continuous peritoneal dialysis" OR "capd" OR "peritoneum dialysis" OR "peritoneal dialysis" OR "crrt" OR "continuous renal replacement therapy" OR "chronic haemodialysis" OR "chronic hemodialysis" OR "chronic intermittent haemodialysis" OR "chronic intermittent hemodialysis" OR "intermittent chronic haemodialysis" OR "intermittent chronic hemodialysis" OR "intermittent haemodialysis" OR "intermittent hemodialysis" OR "renal dialysis" OR "hemodialysis" OR "haemodialysis" OR "hemodialysis center" OR "hemodialysis department" OR "hemodialysis unit" OR "haemodialysis center" OR "haemodialysis centre" OR "haemodialysis department" OR "haemodialysis unit" OR "haemodialysis, home" OR "hemodialysis, home" OR "home haemodialysis" OR "home hemodialysis" OR "home dialysis" OR "artificial kidney" OR "chronic kidney disease" OR "chronic renal failure" OR "chronic renal insufficiency" OR "kidney failure" OR "kidney diseases" OR "renal failure" OR "renal insufficiency" OR "chronic kidney diseases of uncertain etiology") ) |
| S5 | TX allocat* random* OR (MH "Quantitative Studies") OR (MH "Placebos") OR TX placebo* OR TX random* allocat* OR (MH "Random Assignment") OR TX randomi* control* trial* OR TX ( (singl* N1 blind*) or (singl* N1 mask*) ) OR TX ( (doubl* N1 blind*) or (doubl* N1 mask*) ) OR TX ( (tripl* N1 blind*) or (tripl* N1 mask*) ) OR TX ( (trebl* N1 blind*) or (trebl* N1 mask*) ) OR (TX clinic* N1 trial*) OR PT Clinical trial OR (MH "Clinical Trials+") |
| S6 | S1 OR S2 |
| S7 | S3 OR S4 |
| S8 | S5 AND S6 AND S7 |
| Web of science, until 26 July 2024 | |
| #1 | **TS=(telehealth OR telemedicine OR telemedical OR telenursing OR telemonitoring OR telerehabilitation OR telepractice OR telepractic OR telecare OR 'e health' OR (digital AND health) OR (mobile AND health) OR (digital AND interventions) OR 'tele medicine' OR 'virtual medicine' OR 'tele-nursing' OR 'virtual nursing' OR 'Ehealth' OR 'Telemedicina' OR 'Mobile Health' OR 'Remote Consultation')** and **Preprint Citation Index** (Exclude – Database) |
| #2 | **TS=(("ambulatory continuous peritoneal dialysis" OR "capd" OR "peritoneum dialysis" OR "peritoneal dialysis" OR "crrt" OR "continuous renal replacement therapy" OR "chronic haemodialysis" OR "chronic hemodialysis" OR "chronic intermittent haemodialysis" OR "chronic intermittent hemodialysis" OR "intermittent chronic haemodialysis" OR "intermittent chronic hemodialysis" OR "intermittent haemodialysis" OR "intermittent hemodialysis" OR "renal dialysis" OR "hemodialysis" OR "haemodialysis" OR "hemodialysis center" OR "hemodialysis department" OR "hemodialysis unit" OR "haemodialysis center" OR "haemodialysis centre" OR "haemodialysis department" OR "haemodialysis unit" OR "haemodialysis, home" OR "hemodialysis, home" OR "home haemodialysis" OR "home hemodialysis" OR "home dialysis" OR "artificial kidney" OR "chronic kidney disease" OR "chronic renal failure" OR "chronic renal insufficiency" OR "kidney failure" OR "kidney diseases" OR "renal failure" OR "renal insufficiency" OR "chronic kidney diseases of uncertain etiology"))** and **Preprint Citation Index** (Exclude – Database) |
| #3 | **TS=(randomised OR randomized OR randomisation OR randomisation OR placebo* OR (random* AND (allocat* OR assign*) ) OR (blind* AND (single OR double OR treble OR triple) )) NOT TS=(animal or animals or pisces or fish or fishes or catfish or catfishes or sheatfish or silurus or arius or heteropneustes or clarias or gariepinus or fathead minnow or fathead minnows or pimephales or promelas or cichlidae or trout or trouts or char or chars or salvelinus or salmo or oncorhynchus or guppy or guppies or millionfish or poecilia or goldfish or goldfishes or carassius or auratus or mullet or mullets or mugil or curema or shark or sharks or cod or cods or gadus or morhua or carp or carps or cyprinus or carpio or killifish or eel or eels or anguilla or zander or sander or lucioperca or stizostedion or turbot or turbots or psetta or flatfish or flatfishes or plaice or pleuronectes or platessa or tilapia or tilapias or oreochromis or sarotherodon or common sole or dover sole or solea or zebrafish or zebrafishes or danio or rerio or seabass or dicentrarchus or labrax or morone or lamprey or lampreys or petromyzon or pumpkinseed or pumpkinseeds or lepomis or gibbosus or herring or clupea or harengus or amphibia or amphibian or amphibians or anura or salientia or frog or frogs or rana or toad or toads or bufo or xenopus or laevis or bombina or epidalea or calamita or salamander or salamanders or newt or newts or triturus or reptilia or reptile or reptiles or bearded dragon or pogona or vitticeps or iguana or iguanas or lizard or lizards or anguis fragilis or turtle or turtles or snakes or snake or aves or bird or birds or quail or quails or coturnix or bobwhite or colinus or virginianus or poultry or poultries or fowl or fowls or chicken or chickens or gallus or zebra finch or taeniopygia or guttata or canary or canaries or serinus or canaria or parakeet or parakeets or grasskeet or parrot or parrots or psittacine or psittacines or shelduck or tadorna or goose or geese or branta or leucopsis or woodlark or lullula or flycatcher or ficedula or hypoleuca or dove or doves or geopelia or cuneata or duck or ducks or greylag or graylag or anser or harrier or circus pygargus or red knot or great knot or calidris or canutus or godwit or limosa or lapponica or meleagris or gallopavo or jackdaw or corvus or monedula or ruff or philomachus or pugnax or lapwing or peewit or plover or vanellus or swan or cygnus or columbianus or bewickii or gull or chroicocephalus or ridibundus or albifrons or great tit or parus or aythya or fuligula or streptopelia or risoria or spoonbill or platalea or leucorodia or blackbird or turdus or merula or blue tit or cyanistes or pigeon or pigeons or columba or pintail or anas or starling or sturnus or owl or athene noctua or pochard or ferina or cockatiel or nymphicus or hollandicus or skylark or alauda or tern or sterna or teal or crecca or oystercatcher or haematopus or ostralegus or shrew or shrews or sorex or araneus or crocidura or russula or european mole or talpa or chiroptera or bat or bats or eptesicus or serotinus or myotis or dasycneme or daubentonii or pipistrelle or pipistrellus or cat or cats or felis or catus or feline or dog or dogs or canis or canine or canines or otter or otters or lutra or badger or badgers or meles or fitchew or fitch or foumart or foulmart or ferrets or ferret or polecat or polecats or mustela or putorius or weasel or weasels or fox or foxes or vulpes or common seal or phoca or vitulina or grey seal or halichoerus or horse or horses or equus or equine or equidae or donkey or donkeys or mule or mules or pig or pigs or swine or swines or hog or hogs or boar or boars or porcine or piglet or piglets or sus or scrofa or llama or llamas or lama or glama or deer or deers or cervus or elaphus or cow or cows or bos taurus or bos indicus or bovine or bull or bulls or cattle or bison or bisons or sheep or sheeps or ovis aries or ovine or lamb or lambs or mouflon or mouflons or goat or goats or capra or caprine or chamois or rupicapra or leporidae or lagomorpha or lagomorph or rabbit or rabbits or oryctolagus or cuniculus or laprine or hares or lepus or rodentia or rodent or rodents or murinae or mouse or mice or mus or musculus or murine or woodmouse or apodemus or rat or rats or rattus or norvegicus or guinea pig or guinea pigs or cavia or porcellus or hamster or hamsters or mesocricetus or cricetulus or cricetus or gerbil or gerbils or jird or jirds or meriones or unguiculatus or jerboa or jerboas or jaculus or chinchilla or chinchillas or beaver or beavers or castor fiber or castor canadensis or sciuridae or squirrel or squirrels or sciurus or chipmunk or chipmunks or marmot or marmots or marmota or suslik or susliks or spermophilus or cynomys or cottonrat or cottonrats or sigmodon or vole or voles or microtus or myodes or glareolus or primate or primates or prosimian or prosimians or lemur or lemurs or lemuridae or loris or bush baby or bush babies or bushbaby or bushbabies or galago or galagos or anthropoidea or anthropoids or simian or simians or monkey or monkeys or marmoset or marmosets or callithrix or cebuella or tamarin or tamarins or saguinus or leontopithecus or squirrel monkey or squirrel monkeys or saimiri or night monkey or night monkeys or owl monkey or owl monkeys or douroucoulis or aotus or spider monkey or spider monkeys or ateles or baboon or baboons or papio or rhesus monkey or macaque or macaca or mulatta or cynomolgus or fascicularis or green monkey or green monkeys or chlorocebus or vervet or vervets or pygerythrus or hominoidea or ape or apes or hylobatidae or gibbon or gibbons or siamang or siamangs or nomascus or symphalangus or hominidae or orangutan or orangutans or pongo or chimpanzee or chimpanzees or pan troglodytes or bonobo or bonobos or pan paniscus or gorilla or gorillas or troglodytes)** and **Preprint Citation Index** (Exclude – Database) |
| #4 | #1 AND #2 AND #3 |
| Wanfang, until 27 July 2024 | |
| #1 | ((Subject: 透析) OR (Title/Abstract/Keywords: 腹膜透析) OR (Subject: 腹膜透析) OR (Title/Abstract/Keywords: 血液透析) OR (Subject: 血液透析) OR (Title/Abstract/Keywords: 维持性透析) OR (Subject: 维持性透析) OR (Title/Abstract/Keywords: 尿毒症) OR (Subject: 尿毒症) OR (Title/Abstract/Keywords: 透析))  AND  ((Subject: 远程护理) OR (Title/Abstract/Keywords: 远程护理) OR (Subject: 远程医疗) OR (Title/Abstract/Keywords: 远程医疗) OR (Subject: 远程医疗服务) OR (Title/Abstract/Keywords: 远程医疗服务) OR (Keywords: 远程患者管理) OR (Title: 远程患者管理) OR (Subject: 互联网+护理) OR (Title/Abstract/Keywords: 互联网+护理) OR (Subject: 互联网+护理 AND 远程护理) OR (Title/Abstract/Keywords: 互联网+护理 AND 远程护理)) |
| CNKI, until 27 July 2024 | |
| #1 | Dialysis terms (Chinese keywords as used): 透析; 透析患者; 透析治疗; 血液透析; 血液透析患者; 腹膜透析; 维持性; 维持性血液透析; 维持性血液透析患者; 维持性血透; 维持性腹膜透析; 维持性透析 |
| #2 | Telehealth/telenursing terms (Chinese keywords as used): 远程医疗; 远程医疗服务; 远程医疗监护; 远程医疗技术; 远程医疗平台; 远程医疗诊断; 远程护理; 远程护理干预; 远程护理教育; 远程护理模式; 远程护理服务; 远程护理会诊; “互联网+护理服务”; “互联网+护理模式” |
|  | #1 AND #2 |
| Ichushi-Web, until 27 July 2024 | |
| #1 | テレヘルス/AL |
| #2 | (遠隔看護/TH or テレナーシング/AL) |
| #3 | (遠隔看護/TH or テレナーシング/AL) |
| #4 | (遠隔医療/TH or 遠隔医療/AL) |
| #5 | (遠隔看護/TH or 遠隔看護/AL) |
| #6 | #1 or #2 or #3 or #4 or #5 |
| #7 | (血液透析/TH or 透析/AL) |
| #8 | (血液透析/TH or 透析/AL) |
| #9 | (腹膜透析/TH or 腹膜透析/AL) |
| #10 | (腹膜透析/TH or 腹膜透析/AL) |
| #11 | #7 or #8 or #9 or #10 |
| #12 | #6 and #11 |
| #13 | (腹膜透析/TH or 腹膜透析/AL) |
| #14 | #12 and #13 |
| #15 | (#14) and (Publication type = Original article) |
